# Supplementary material for: Hot spot prediction in protein-protein interactions by an ensemble system
Source: BMC Syst Biol. 2018 Dec 31;12(Suppl 9):132. doi: 10.1186/s12918-018-0665-8 (PMC6311905; doi:10.1186/s12918-018-0665-8)
Supplement: Supplementary file 2 — All relevant MATLAB code. A simple Matlab implement of our predictor. (ZIP 19,742 kb) [file 12918_2018_665_MOESM2_ESM.zip › Additional file 2/Read ASA/ASA Property/SKEMPI/1JRHL.txt]

 NetSurfP - Protein Surface Accessibility and Secondary Structure Predictions

  

|  |  |  |
| --- | --- | --- |
|  |  | NetSurfP - Protein Surface Accessibility and Secondary Structure Predictions  Technical University of Denmark |

  


---

```
# For publication of results, please cite:
# A generic method for assignment of reliability scores applied to solvent accessibility predictions.
# Bent Petersen, Thomas Nordahl Petersen, Pernille Andersen, Morten Nielsen and Claus Lundegaard
# BMC Structural Biology 2009, 9:51 doi:10.1186/1472-6807-9-51
#
# Column 1: Class assignment - B for buried or E for Exposed - Threshold: 25% exposure, but not based on RSA
# Column 2: Amino acid
# Column 3: Sequence name
# Column 4: Amino acid number
# Column 5: Relative Surface Accessibility - RSA
# Column 6: Absolute Surface Accessibility
# Column 7: Z-fit score for RSA prediction
# Column 8: Probability for Alpha-Helix
# Column 9: Probability for Beta-strand
# Column 10: Probability for Coil
E S  1JRHL                  1    0.727  85.251  -0.076   0.005   0.015   0.979
B V  1JRHL                  2    0.291  44.788  -0.282   0.003   0.718   0.279
E E  1JRHL                  3    0.506  88.381   1.399   0.001   0.900   0.099
B M  1JRHL                  4    0.143  28.654   0.554   0.001   0.900   0.099
E T  1JRHL                  5    0.448  62.193   0.155   0.003   0.718   0.279
B Q  1JRHL                  6    0.151  26.915   0.329   0.004   0.420   0.576
E S  1JRHL                  7    0.386  45.216   0.279   0.004   0.085   0.910
E P  1JRHL                  8    0.471  66.807  -0.785   0.018   0.019   0.964
E S  1JRHL                  9    0.540  63.300  -0.998   0.052   0.084   0.864
B S  1JRHL                 10    0.323  37.809  -1.037   0.069   0.386   0.545
B F  1JRHL                 11    0.198  39.638  -0.017   0.021   0.756   0.223
E S  1JRHL                 12    0.393  46.106  -0.526   0.021   0.756   0.223
B V  1JRHL                 13    0.213  32.723  -0.122   0.023   0.655   0.322
E S  1JRHL                 14    0.420  49.224   0.593   0.021   0.279   0.699
E L  1JRHL                 15    0.494  90.360   0.465   0.004   0.085   0.910
E G  1JRHL                 16    0.570  44.843  -1.407   0.005   0.045   0.951
E D  1JRHL                 17    0.442  63.707   0.472   0.004   0.138   0.858
E R  1JRHL                 18    0.542 124.026   1.093   0.003   0.718   0.279
B V  1JRHL                 19    0.064   9.852   0.271   0.001   0.900   0.099
E T  1JRHL                 20    0.411  57.061   1.237   0.001   0.959   0.040
B I  1JRHL                 21    0.051   9.379   0.364   0.001   0.959   0.040
E T  1JRHL                 22    0.368  51.083   0.814   0.001   0.959   0.040
B C  1JRHL                 23    0.028   3.875   0.272   0.001   0.900   0.099
E K  1JRHL                 24    0.405  83.350   0.969   0.001   0.900   0.099
B A  1JRHL                 25    0.237  26.172  -1.192   0.002   0.816   0.182
B S  1JRHL                 26    0.338  39.660  -1.164   0.004   0.616   0.381
E E  1JRHL                 27    0.512  89.394  -2.059   0.004   0.197   0.799
E D  1JRHL                 28    0.430  61.920  -2.395   0.018   0.047   0.935
E I  1JRHL                 29    0.440  81.418  -1.733   0.004   0.138   0.858
E Y  1JRHL                 30    0.362  77.338  -1.332   0.019   0.141   0.840
B N  1JRHL                 31    0.312  45.750  -0.981   0.005   0.262   0.733
B R  1JRHL                 32    0.134  30.571   0.695   0.004   0.616   0.381
B L  1JRHL                 33    0.028   5.127   0.384   0.018   0.846   0.136
B A  1JRHL                 34    0.083   9.191   0.252   0.011   0.918   0.071
B W  1JRHL                 35    0.028   6.638   0.619   0.011   0.918   0.071
B Y  1JRHL                 36    0.038   8.078   0.523   0.011   0.918   0.071
B Q  1JRHL                 37    0.128  22.843   0.650   0.018   0.846   0.136
B Q  1JRHL                 38    0.086  15.431   0.766   0.023   0.655   0.322
E K  1JRHL                 39    0.394  81.066   1.707   0.004   0.085   0.910
E P  1JRHL                 40    0.445  63.117  -0.276   0.005   0.015   0.979
E G  1JRHL                 41    0.758  59.670  -2.185   0.003   0.003   0.994
E N  1JRHL                 42    0.437  63.918   0.087   0.018   0.019   0.964
E A  1JRHL                 43    0.299  32.950  -0.402   0.018   0.047   0.935
B P  1JRHL                 44    0.086  12.260   0.322   0.005   0.262   0.733
B R  1JRHL                 45    0.255  58.349   0.687   0.002   0.816   0.182
B L  1JRHL                 46    0.098  17.907   0.619   0.001   0.900   0.099
B L  1JRHL                 47    0.034   6.152   0.512   0.001   0.959   0.040
B I  1JRHL                 48    0.017   3.163   1.161   0.001   0.959   0.040
B S  1JRHL                 49    0.076   8.872   0.143   0.000   0.983   0.017
B G  1JRHL                 50    0.090   7.114  -0.048   0.001   0.959   0.040
B A  1JRHL                 51    0.146  16.067  -0.359   0.003   0.718   0.279
B T  1JRHL                 52    0.195  27.088  -0.806   0.004   0.420   0.576
E S  1JRHL                 53    0.574  67.261  -1.313   0.019   0.141   0.840
E L  1JRHL                 54    0.435  79.630  -0.707   0.005   0.045   0.951
E E  1JRHL                 55    0.656 114.533  -1.813   0.018   0.047   0.935
E T  1JRHL                 56    0.681  94.413  -1.419   0.018   0.047   0.935
E E  1JRHL                 57    0.632 110.410  -1.217   0.018   0.088   0.893
E V  1JRHL                 58    0.308  47.324  -0.101   0.004   0.085   0.910
E P  1JRHL                 59    0.545  77.307  -1.121   0.004   0.085   0.910
E S  1JRHL                 60    0.385  45.157  -1.702   0.004   0.197   0.799
B R  1JRHL                 61    0.218  49.968  -0.741   0.004   0.616   0.381
B F  1JRHL                 62    0.071  14.169  -0.918   0.002   0.816   0.182
B S  1JRHL                 63    0.192  22.561  -0.681   0.001   0.900   0.099
B G  1JRHL                 64    0.093   7.303  -2.091   0.002   0.816   0.182
E S  1JRHL                 65    0.395  46.294   0.091   0.004   0.616   0.381
E G  1JRHL                 66    0.407  31.999  -0.585   0.005   0.336   0.660
E S  1JRHL                 67    0.598  70.109  -1.569   0.005   0.045   0.951
E G  1JRHL                 68    0.652  51.336  -1.110   0.005   0.015   0.979
E K  1JRHL                 69    0.420  86.312  -0.154   0.004   0.197   0.799
E D  1JRHL                 70    0.400  57.582   0.377   0.003   0.718   0.279
B Y  1JRHL                 71    0.065  13.976  -0.105   0.001   0.900   0.099
B T  1JRHL                 72    0.207  28.766   0.559   0.001   0.900   0.099
B L  1JRHL                 73    0.032   5.878   0.238   0.001   0.959   0.040
E S  1JRHL                 74    0.282  33.039   1.155   0.001   0.900   0.099
B I  1JRHL                 75    0.057  10.600   0.188   0.002   0.816   0.182
E T  1JRHL                 76    0.459  63.608   0.233   0.022   0.552   0.426
E S  1JRHL                 77    0.459  53.736  -0.415   0.022   0.359   0.619
B L  1JRHL                 78    0.101  18.548   0.239   0.020   0.205   0.775
E Q  1JRHL                 79    0.407  72.780   0.838   0.019   0.141   0.840
E T  1JRHL                 80    0.440  61.083  -0.496   0.018   0.088   0.893
E E  1JRHL                 81    0.566  98.950  -1.178   0.053   0.043   0.903
E D  1JRHL                 82    0.375  54.081  -0.972   0.018   0.047   0.935
E V  1JRHL                 83    0.400  61.419  -0.559   0.019   0.141   0.840
B A  1JRHL                 84    0.159  17.544  -1.077   0.021   0.451   0.528
B T  1JRHL                 85    0.236  32.719  -0.147   0.023   0.655   0.322
B Y  1JRHL                 86    0.052  11.006   0.556   0.021   0.756   0.223
B Y  1JRHL                 87    0.065  13.869   0.735   0.021   0.756   0.223
B C  1JRHL                 88    0.022   3.089   0.635   0.023   0.655   0.322
B Q  1JRHL                 89    0.039   6.912  -0.712   0.022   0.552   0.426
B Q  1JRHL                 90    0.069  12.359  -1.210   0.022   0.552   0.426
B Y  1JRHL                 91    0.198  42.355  -1.085   0.005   0.336   0.660
E W  1JRHL                 92    0.433 104.064  -1.652   0.004   0.085   0.910
B S  1JRHL                 93    0.324  37.961  -1.854   0.005   0.015   0.979
E T  1JRHL                 94    0.366  50.723  -1.727   0.018   0.047   0.935
E W  1JRHL                 95    0.332  79.750  -1.124   0.018   0.088   0.893
B T  1JRHL                 96    0.179  24.883  -1.411   0.020   0.205   0.775
B F  1JRHL                 97    0.116  23.361  -1.305   0.004   0.197   0.799
B G  1JRHL                 98    0.137  10.750  -2.966   0.004   0.085   0.910
E G  1JRHL                 99    0.343  27.033  -0.798   0.004   0.085   0.910
B G  1JRHL                100    0.118   9.302  -2.088   0.004   0.197   0.799
B T  1JRHL                101    0.088  12.206  -0.335   0.004   0.420   0.576
E K  1JRHL                102    0.324  66.544   0.252   0.002   0.816   0.182
B L  1JRHL                103    0.092  16.845  -0.402   0.002   0.816   0.182
E E  1JRHL                104    0.357  62.368   0.617   0.021   0.756   0.223
B I  1JRHL                105    0.143  26.474  -0.774   0.023   0.655   0.322
E K  1JRHL                106    0.522 107.273  -0.434   0.004   0.514   0.481
E R  1JRHL                107    0.439 100.577  -0.648   0.021   0.279   0.699
E A  1JRHL                108    0.639  70.462  -0.537   0.018   0.088   0.893
E D  1JRHL                109    0.549  79.068  -1.114   0.018   0.047   0.935
E A  1JRHL                110    0.538  59.343  -1.347   0.018   0.047   0.935
E A  1JRHL                111    0.568  62.638  -1.102   0.018   0.019   0.964
E P  1JRHL                112    0.338  47.891  -0.482   0.018   0.019   0.964
E T  1JRHL                113    0.471  65.383   0.333   0.004   0.085   0.910
B V  1JRHL                114    0.111  17.107  -0.276   0.005   0.336   0.660
B S  1JRHL                115    0.141  16.502  -0.656   0.003   0.718   0.279
B I  1JRHL                116    0.114  21.090  -1.090   0.002   0.816   0.182
B F  1JRHL                117    0.068  13.567  -0.095   0.003   0.718   0.279
B P  1JRHL                118    0.079  11.153   0.271   0.022   0.359   0.619
B C  1JRHL                119    0.034   4.830  -0.477   0.022   0.359   0.619
B F  1JRHL                120    0.122  24.526  -0.506   0.022   0.552   0.426
B L  1JRHL                121    0.045   8.221  -1.033   0.021   0.451   0.528
E N  1JRHL                122    0.355  51.928   0.086   0.021   0.279   0.699
E N  1JRHL                123    0.482  70.609  -1.700   0.004   0.138   0.858
B F  1JRHL                124    0.271  54.430  -1.368   0.004   0.085   0.910
E Y  1JRHL                125    0.366  78.214  -0.929   0.005   0.045   0.951
E P  1JRHL                126    0.340  48.189  -1.339   0.005   0.015   0.979
E K  1JRHL                127    0.653 134.302  -0.705   0.004   0.085   0.910
E D  1JRHL                128    0.511  73.606  -0.036   0.005   0.336   0.660
B I  1JRHL                129    0.065  12.044   0.334   0.002   0.816   0.182
E N  1JRHL                130    0.364  53.319   0.893   0.001   0.900   0.099
B V  1JRHL                131    0.055   8.530   0.802   0.001   0.959   0.040
E K  1JRHL                132    0.295  60.764   1.008   0.001   0.959   0.040
B G  1JRHL                133    0.102   7.996   0.697   0.001   0.900   0.099
B V  1JRHL                134    0.267  41.084   0.471   0.001   0.900   0.099
E L  1JRHL                135    0.383  70.182  -0.147   0.003   0.718   0.279
E N  1JRHL                136    0.618  90.402  -1.393   0.019   0.141   0.840
E S  1JRHL                137    0.560  65.609  -1.855   0.018   0.088   0.893
E W  1JRHL                138    0.579 139.370  -0.642   0.004   0.420   0.576
E T  1JRHL                139    0.560  77.700   0.447   0.022   0.552   0.426
E D  1JRHL                140    0.344  49.628   0.377   0.022   0.552   0.426
E Q  1JRHL                141    0.507  90.586   0.014   0.022   0.552   0.426
E D  1JRHL                142    0.598  86.114  -0.078   0.022   0.359   0.619
E S  1JRHL                143    0.489  57.264  -0.714   0.004   0.138   0.858
E K  1JRHL                144    0.720 148.186   0.102   0.004   0.085   0.910
E D  1JRHL                145    0.765 110.294  -0.112   0.005   0.045   0.951
E S  1JRHL                146    0.319  37.375  -0.989   0.004   0.085   0.910
E T  1JRHL                147    0.396  54.939   1.039   0.004   0.616   0.381
B Y  1JRHL                148    0.116  24.768   0.063   0.001   0.900   0.099
B S  1JRHL                149    0.120  14.017  -0.668   0.001   0.900   0.099
B M  1JRHL                150    0.074  14.747  -0.284   0.001   0.900   0.099
B S  1JRHL                151    0.069   8.099  -1.214   0.001   0.959   0.040
B S  1JRHL                152    0.040   4.723  -0.564   0.001   0.959   0.040
B T  1JRHL                153    0.229  31.776   0.319   0.001   0.959   0.040
B C  1JRHL                154    0.032   4.437   0.044   0.001   0.959   0.040
E E  1JRHL                155    0.311  54.279   0.714   0.001   0.900   0.099
B A  1JRHL                156    0.056   6.204   0.751   0.003   0.718   0.279
E T  1JRHL                157    0.387  53.649   1.587   0.004   0.420   0.576
E H  1JRHL                158    0.386  70.213  -0.191   0.019   0.141   0.840
E K  1JRHL                159    0.678 139.465  -0.523   0.052   0.084   0.864
E T  1JRHL                160    0.545  75.564  -0.070   0.052   0.084   0.864
B S  1JRHL                161    0.152  17.826  -0.585   0.053   0.043   0.903
E T  1JRHL                162    0.632  87.714   0.294   0.053   0.043   0.903
E S  1JRHL                163    0.307  35.969  -0.561   0.018   0.019   0.964
E P  1JRHL                164    0.457  64.848  -0.600   0.018   0.047   0.935
E I  1JRHL                165    0.312  57.628  -1.593   0.018   0.088   0.893
E V  1JRHL                166    0.556  85.519   0.376   0.019   0.141   0.840
E K  1JRHL                167    0.801 164.869   0.575   0.003   0.003   0.994

Explain the output.  Go back.
```

---
